# A Rare Case of Triple Neuromas As a Cause of Chronic Stump Pain in an Upper Limb Amputee

**Published:** 2016-03-12

**Authors:** M. Chasapi, A. Salibi, R. Wain, S. Iyer

**Affiliations:** Plastic Surgery Department, Royal Preston Hospital, Sharoe Green Lane, Preston, Lancashire, England

**Keywords:** amputation neuroma, neuroma, centrocentral nerve repair, chronic stump pain, through-wrist amputation

## DESCRIPTION

**Figure 1:**
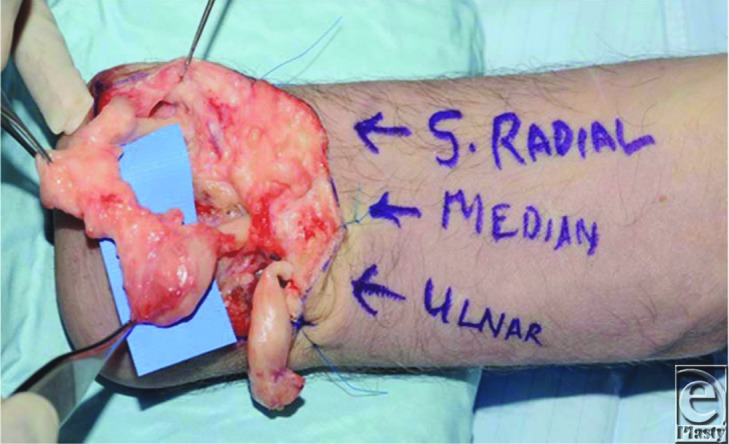
During the exploration, large neuromas were identified to the ulnar, median, and superficial radial nerves.

A 54-year-old man with through-wrist amputation of his right-dominant hand presented with chronic stump pain that was resistant to conservative treatment. Clinical examination was compatible with the presence of neuroma. Surgical exploration revealed neuromas of the ulnar, median, and superficial radial nerve stumps (Fig 1).

## QUESTIONS

1. **What is neuroma, how is it formed, and what are the symptoms?**
2. **What is the significance of amputation neuromas?**
3. **What is the management of painful neuromas, and what methods of surgical treatment are described?**
4. **What is the evidence behind nerve-to-nerve repair?**

## DISCUSSION

The term “neuroma” refers to a disorganized fibroneural mass containing peripheral nerve components such as axons, connective tissue, and a variety of cells including Schwann cells, macrophages, and myofibroblasts. Neuromas are formed after peripheral nerve injury as a result of ineffective, unregulated, and abnormal regeneration of the sprouting axons.^1^^,^^2^ The exact incidence of postoperative neuromas is unknown because most are asymptomatic. Only a small proportion of those containing sensory nerve fibers will become painful and clinically pathological. The reported incidence of symptomatic painful neuromas ranges between 1% and 30%.^1^

In most cases, the diagnosis of neuroma is made clinically.^3^ The pain is usually localized and described as sharp, shooting, or electric shock-like sensation, which is exacerbated by prosthesis wear, weight bearing, or mechanical pressure.

Amputation neuromas are often overlooked, as there are a multitude of other potential causes for stump pain in amputees such as phantom pain, joint problems, ischemic pain, referred pain, and poor prosthetic fit.^3^ However, between 10% and 25% of patients with chronic stump pain are found to have a neuroma.^4^ Chronic stump pain can be a major issue for this patient group, causing significant distress, affecting quality of life, and hindering rehabilitation and subsequent prosthetic use.^3^

**Figure 2:**
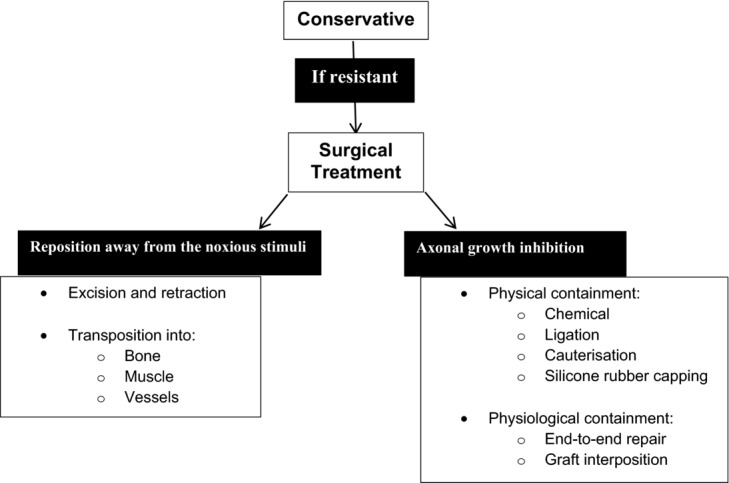
Painful neuromas management algorithm. From Geraghty and Jones,^3^ Wood and Mudge,^5^ and Low et al.^7^

The initial management of a painful neuroma is usually conservative, with reservation of surgical intervention for intractable cases. 3^,^^4^ Multiple surgical techniques have been described, attempting to either reposition the stump away from the noxious stimuli (translocation) or inhibit the axonal growth.^3^^,^^5^^-^8 Translocation may either involve excision and retraction or involve transposition into muscle, bone, or vessels. Axonal growth inhibition can be attempted either by physical containment such as chemical treatment, ligation, cauterization, and silicone capping or by physiological containment such as nerve-to-nerve repair techniques (Fig 2).^3^^,^^5^^-^8

Nerve-to-nerve repair techniques appear to be the most successful treatment^7^ and may involve interposition of a nerve graft between the 2 nerve ends. The coaptation of 2 nerve cords of central origin is called centrocentral nerve repair.^5^^-^8 It is based on Langley and Anderson's observation that regenerating axons would not grow into endoneural tubes that are already occupied by axons.^5^^-^8 This is because the approaching neurons push axoplasm toward each other, thus increasing the intraneural pressure that, in turn, inhibits central protein synthesis and leads to neuroma development.^7^ The end-to-end connection across interposed graft between 2 nerve cords of central origin is called centrocentral nerve union or centrocentralization with autologous transplantation and was first introduced by Gorkish.^6^^-^8 This technique works by the combined effect of (*a*) isolating the proximal segment from the target-derived neuronotrophic factors and (*b*) confining them in a nontarget environment, in addition to the increased endoneural pressure effect seen in the nongraft technique.^6^^-^8 Centrocentral union with autologous graft has proven effective both in the prevention of finger stump neuromas and in lower extremity amputation stump neuromas.^6^^-^8 However, the current literature lacks enough evidence regarding its efficacy in forearm or wrist amputation neuromas, where the direct primary coaptation of 2 distal nerve ends without interposition of an autologous nerve graft seems to be more effective.^5^^,^^8^

**Figure 3:**
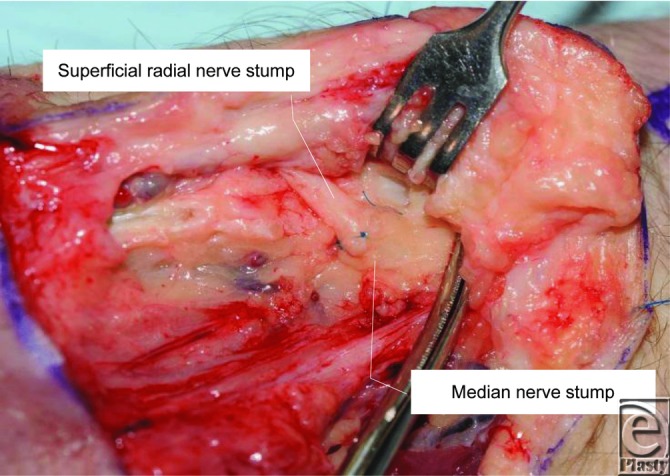
End-to-end epineural anastomosis was performed between the median and superficial radial nerve stumps.

On this basis and taking into account that neuromas of the superficial radial nerve have often been described as one of the more difficult neuromas to manage,^6^ we proceeded with a direct, end-to-end neurosynthesis of the median and superficial radial nerve ends (Fig 3), whereas the ulnar nerve was left to retract. The patient's neuroma-related pain was reduced by 90% postoperatively with full recovery.
